# Special Issue “Proteomics: An Invaluable Tool to Gain Information on Biological Systems and Their Disease Alterations”

**DOI:** 10.3390/ijms262211197

**Published:** 2025-11-19

**Authors:** Paolo Iadarola, Simona Viglio

**Affiliations:** 1 Department of Biology and Biotechnology “L. Spallanzani”, University of Pavia, 27100 Pavia, Italy; 2 Unit of Biochemistry, Department of Molecular Medicine, University of Pavia, 27100 Pavia, Italy; simona.viglio@unipv.it

Over the past two decades, proteomics has evolved from a descriptive cataloging of proteins to a sophisticated analytical science that provides deep insights into the dynamic complexity of biological systems. The proteome is the functional translation of the genome and reflects not only genetic information but also environmental influences, post-translational modifications, and molecular interactions that together determine cellular behavior. Consequently, proteomic technologies have become indispensable tools for understanding physiological and pathological processes at a systems level [1]. Recent advances in high-resolution mass spectrometry, quantitative labeling strategies, and bioinformatic pipelines have dramatically increased the depth, accuracy, and reproducibility of proteomic analyses. Today, from limited sample amounts, it is possible to profile thousands of proteins and their modifications, monitor temporal changes, and integrate multi-omics data to achieve a truly holistic view of biology. These developments have strengthened the role of proteomics as a central pillar of systems biology and precision medicine [1]. In the context of disease, proteomic investigations offer unique advantages. While enabling the identification of biomarkers for early diagnosis, patient stratification, and therapeutic monitoring, they also clarify the molecular mechanisms underlying pathological states. For instance, differential proteomic studies have revealed disease-specific expression patterns in cancer, neurodegenerative, cardiovascular, and metabolic disorders, uncovering previously unrecognized regulatory pathways and potential drug targets [2]. Moreover, spatial and single-cell proteomics are now shedding light on tissue microenvironments and cellular heterogeneity, areas traditionally inaccessible to bulk analyses [3]. An equally important frontier lies in the study of extracellular vesicles, secretomes, and biofluids, which provide minimally invasive access to disease-related proteomes [4]. These circulating protein signatures hold promise for the development of non-invasive diagnostic and prognostic tools, opening new perspectives for translational research and clinical application [5].

This Special Issue gathers contributions that exemplify the power and versatility of proteomic approaches. The articles collected here explore diverse biological contexts, from fundamental mechanisms of cellular regulation to complex disease models and highlight both methodological innovations and conceptual advances. Together, they underscore how proteomics continues to expand the boundaries of biomedical knowledge, fostering connections between basic research and clinical practice.

It is expected that the studies presented in this Special Issue will encourage further exploration into the proteome’s dynamic landscape. The integration of proteomics with genomics, metabolomics, lipidomics, and transcriptomics will undoubtedly accelerate the transition from descriptive biology to predictive and personalized medicine. Ultimately, the continued evolution of proteomic science promises not only to deepen our understanding of biological systems but also to translate this knowledge into tangible benefits for human health.
